# Weight changes and all-cause mortality in critically ill patients: a multi-center retrospective cohort study

**DOI:** 10.3389/fnut.2026.1722116

**Published:** 2026-05-11

**Authors:** Cheng Luo, Tinglong Zhang, Xiaoyong Xiao, Zhe Deng

**Affiliations:** 1 Department of Emergency Medicine, Shenzhen Second People’s Hospital, The First Affiliated Hospital of Shenzhen University, Shenzhen, China; 2 Department of Emergency Medicine, South China Hospital Affiliated to Shenzhen University, Shenzhen, China

**Keywords:** critically ill patients, eICU database, mortality, retrospective cohort study, weight change rate

## Abstract

**Objective:**

Body weight changes is a readily available composite metric in critically ill patients that may reflect both fluid balance and nutritional-metabolic status. However, large-scale evidence on its independent association with—and predictive value for—patient-centered outcomes such as mortality remains limited.

**Methods:**

This large, multicenter retrospective study used the U.S. eICU Collaborative Research Database. A total of 30,537 adult critically ill patients were included. The primary exposure was weight change rate. Feature selection was performed with the Boruta machine-learning algorithm. Multivariable logistic regression was used to estimate the association between weight change rate and mortality. Non-linear relationships and threshold effects were explored with generalized additive models (GAM) and two-piece linear regression. Discriminative performance was assessed with the area under the receiver operating characteristic curve (AUC).

**Results:**

Boruta identified body weight change rate as an important predictor of both ICU and hospital mortality. After multivariable adjustment, each additional percentage of weight gain was associated with a significant increase in ICU mortality (OR 1.04, 95% CI 1.03–1.05) and hospital mortality (OR 1.03, 95% CI 1.03–1.04) (both *p* < 0.0001). The association between weight change rate and mortality risk was non-linear, with a slope change (threshold effect) occurring at approximately 5% (ICU mortality: 5.14%; hospital mortality: 4.73%). Weight change rate provided superior discrimination compared with static indices such as admission weight, discharge weight, or admission BMI.

**Conclusion:**

In this large cohort, the ICU-derived weight change rate is an independent predictor of mortality in critically ill patients and exhibits a non-linear relationship with a threshold effect at approximately 5%. Dynamic monitoring of weight change rate outperforms static anthropometric measures and may serve as a simple yet powerful bedside tool for prognostic assessment.

## Introduction

Nutritional status and its dynamic trajectory are increasingly recognized as pivotal determinants of clinical outcomes in critically ill patients (1). The catabolic stress response triggered by critical illness precipitates rapid muscle wasting and metabolic derangements, both of which are strongly associated with increased morbidity and mortality (2). However, body-composition metrics such as sarcopenic obesity more accurately reflect nutritional state (3). While traditional intensive-care nutrition has focused on delivering adequate calories and protein, there is growing interest in simple, objective, and readily accessible biomarkers that reflect a patient’s metabolic and fluid status and can reliably predict prognosis.

Body weight is a fundamental anthropometric parameter. In the highly dynamic ICU environment, weight change is a composite metric shaped by the complex interplay of fluid resuscitation, capillary leak, nutritional intake, catabolism, and diuretic use. Substantial weight gain most often signals fluid overload, an independent contributor to organ dysfunction, prolonged mechanical ventilation, and death (4, 5). Conversely, rapid weight loss may indicate severe catabolism and muscle depletion—i.e., ICU-acquired weakness—which powerfully predicts long-term functional impairment and mortality (6). Despite this pathophysiological rationale, the precise role of in-ICU weight change as an independent predictor of patient-centered outcomes remains incompletely defined. Most prior work has centered on static indices such as admission weight or body-mass index (BMI), yet their association with mortality in this heterogeneous population has yielded inconsistent findings (7, 8). Although studies such as You et al. (9) and Zhang et al. (10) reported links between weight or BMI change and clinical outcomes, and Ayalon et al. (11) explored weight as a mortality risk factor in pediatric critical illness, results are heterogeneous and large-scale validation is lacking.

The eICU Collaborative Research Database, a multicenter ICU repository, offers a unique opportunity to investigate these relationships with high statistical power (12). Advanced machine-learning techniques, exemplified by the Boruta algorithm, provide a robust framework for identifying truly informative predictors from a high-dimensional covariate space, mitigating overfitting and highlighting novel prognostic factors (13).

We sought to establish evidence for monitoring weight change as a simple yet powerful bedside prognostic tool, acknowledging it is a surrogate rather than a direct measure of nutrition. We hypothesized that greater weight change, irrespective of direction, would be independently associated with increased mortality. Using rigorous feature selection and multivariable modeling, we sought to establish evidence for monitoring weight change as a simple yet powerful bedside prognostic tool.

## Methods

### Study design and data source and ethics statement

We conducted a large-scale, multicenter, retrospective cohort study using the eICU Collaborative Research Database (eICU-CRD). Assembled in 2014–2015, the eICU-CRD remains the only publicly available U.S. multicenter ICU dataset that contains both admission and discharge body-weight records. Covering >200,000 admissions to >200 ICUs nationwide, the fully de-identified repository was built under a uniform Philips data dictionary and validation scripts; every ICU must pass automated checks for field completeness and logical consistency before upload (12). The study was reported in accordance with the STROBE statement. Because the data are anonymized and in the public domain, the MIT Institutional Review Board deemed the protocol exempt from full review and waived the requirement for informed consent.

### Study population

**Figure 1:**
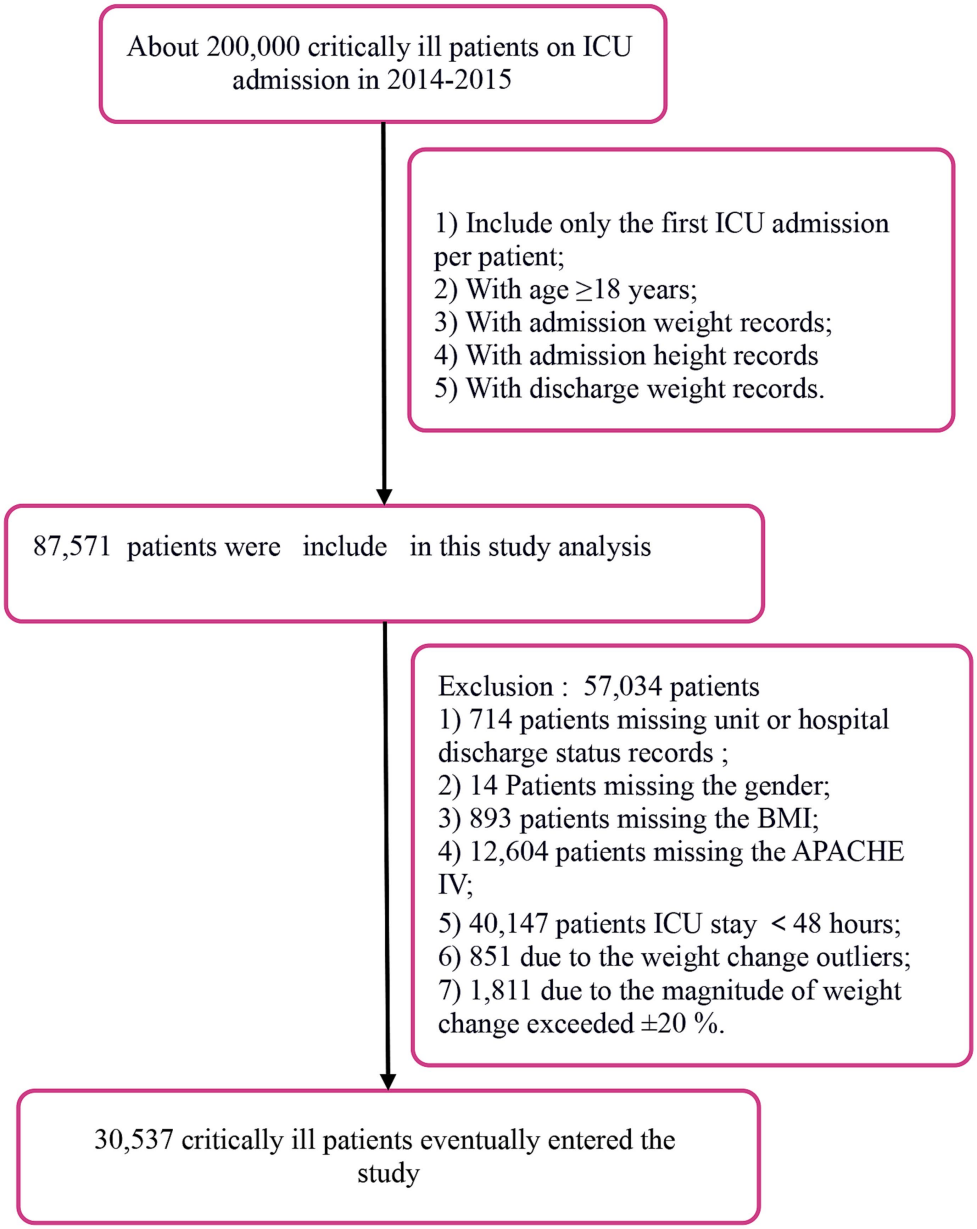
Flow chart of study population. BMI, body mass index; ICU, intensive care unit; APACHE, acute physiology and chronic health evaluation.

We restricted the analysis to the first ICU admission recorded between 2014 and 2015 for patients aged ≥18 years (mean age 64.10 ± 15.99 years), a cohort predominantly composed of middle-aged and older critically ill individuals. The primary exposure was body weight change rate, calculated as (discharge weight − admission weight)/admission weight × 100%. To ensure data quality we sequentially excluded patients with: (1) missing admission or discharge weight; (2) missing gender, BMI or APACHE IV score; (3) ICU stay < 48 h; and (4) absolute weight-change outliers or magnitude > ± 20%. After these exclusions, 30,537 critically ill patients remained for the final analysis; the selection flow is shown in Figure 1.

### Variable definitions

Data were extracted from the eICU-CRD using PostgreSQL (version 17.7) and Navicat Premium (version 16). The extracted variables included the following:

Demographics: Age, sex, and ethnicity. Baseline health status: Body mass index (BMI) at admission, and comorbidities including chronic obstructive pulmonary disease (COPD), heart failure (HF), acute myocardial infarction (AMI), and diabetes mellitus. Illness severity: Acute Physiology and Chronic Health Evaluation IV (APACHE IV) score. ICU interventions: Endotracheal intubation and renal replacement therapy. Hospital course: Length of stay in the ICU and total hospital length of stay. These variables were considered as potential confounders or effect modifiers based on clinical relevance and prior literature.

### Predictor and outcomes

#### Exposure (predictor)

The primary exposure was the body weight change rate during hospitalization, calculated as: [(Discharge weight (kg) − Admission weight (kg))/Admission weight (kg)] × 100%. For each patient, admission and discharge body weights were recorded on the same unit, with the same device, and by the same staff. This continuous variable represents the percentage change from baseline; a positive value indicates weight gain, and a negative value indicates weight loss. It serves as a readily accessible, integrative clinical marker reflecting both metabolic stress and fluid-nutritional dynamics, though the precise contributions of fluid shifts versus lean tissue changes cannot be delineated with these data (14).

#### Outcomes

The primary outcome was all-cause mortality during the intensive care unit stay (ICU mortality). The secondary outcome was all-cause mortality during the entire hospitalization (hospital mortality).

### Statistical analysis

Continuous variables are presented as mean ± standard deviation (SD) or median (interquartile range, IQR), and were compared across tertiles of weight change rate using one-way ANOVA or the Kruskal–Wallis test, as appropriate. Categorical variables are summarized as counts (percentages) and compared using the χ^2^ test.

To identify robust predictors, we applied the Boruta random-forest algorithm separately for ICU and hospital mortality, which ranks variable importance against random “shadow” features to control for noise (13). Subsequently, logistic regression models were constructed in four hierarchical stages: Model I: Unadjusted. Model II: Adjusted for demographics (gender, age, ethnicity, admission BMI). Model III: Additionally adjusted for illness severity (APACHE IV score). Model IV: Fully adjusted for demographics, APACHE IV score, comorbidities (COPD, HF, AMI, diabetes), renal function, and key interventions (endotracheal intubation, renal replacement therapy).

Potential non-linear relationships were explored using generalized additive models (GAM) with smooth terms. A two-piece linear regression model was then fitted to estimate any threshold (knot) effect, the significance of which was tested by the likelihood-ratio test (15). Prespecified subgroup analyses were conducted to assess the consistency of the association across strata of age, sex, BMI, ethnicity, ICU type, discharge year, and APACHE IV score (tertiles). In these analyses, all models were fully adjusted except for the stratification variable itself. The discriminative ability of the weight-change rate was evaluated by calculating the area under the receiver operating characteristic curve (AUC) and compared with that of static weight-related indices (admission weight, discharge weight, admission BMI) for both mortality endpoints.

All analyses were performed using R software (version 4.1.0) with the packages EmpowerStats (www.empowerstats.com; X&Y Solutions, Inc., Boston, MA) and FreeStats. The eICU-CRD data are publicly accessible at https://eicu-crd.mit.edu/, enabling full replication of this study. A two-sided *p*-value <0.05 was considered statistically significant.

## Results

A total of 30,537 critically ill patients were included in the final analytic cohort. According to tertiles of weight change rate, patients were classified as T1 (*n* = 10,179), T2 (*n* = 10,179), and T3 (*n* = 10,179). Baseline characteristics are summarized in Table 1. The three groups differed significantly with respect to age, BMI, gender, ethnicity, and the prevalence of COPD, AMI, diabetes, tracheal intubation, and dialysis (all *p* < 0.001). Heart failure (HF) was the most prevalent condition (4,218 patients [13.8%], identified via ICD-9/10 codes and APACHE flags). We identified 536 cases of Type 4 CRS (pre-existing CKD with concurrent HF). The remaining 3,682 HF patients were classified as unspecified CRS subtype due to database limitations. Renal function markers differed significantly across tertiles, with T3 (weight gain) exhibiting the poorest kidney function [highest Scr and lowest eGFR] compared to T2 (stable weight), which showed relatively preserved renal function (both *p* < 0.001). Notably, BNP levels were comparable among the three groups (*p* = 0.597). Notably, patients in T3, who exhibited the greatest weight gain, had the highest illness severity (APACHE IV score 66.76 ± 27.24), ICU mortality (9.63%), and hospital mortality (13.61%); all comparisons with T1 and T2 were significant (*p* < 0.001). The overall distribution of weight change rate was approximately normal (Supplementary Figure S1).

**Table 1:** The baseline characteristics of participants.

Weight change rate (%)	T1 (−19.98 ~ −0.88)	T2 (−0.88 ~ 2.64)	T3 (2.64 ~ 20.00)	*P*-value
*N*	10,179	10,179	10,179	
Age (year)	63.65 ± 15.98	63.60 ± 16.08	65.05 ± 15.88	<0.001
Admission weight (kg)	85.00 (70.81–103.10)	83.46 (68.90–100.75)	77.10 (63.60–92.70)	<0.001
Discharge weight (kg)	80.00 (66.60–97.33)	83.90 (69.40–101.60)	83.32 (69.08–99.80)	<0.001
BMI (kg/m^2^)	30.95 ± 9.18	30.09 ± 8.55	28.25 ± 8.01	<0.001
Gender, *n* (%)				<0.001
Female	4,275 (42.00%)	4,530 (44.50%)	5,073 (49.84%)	
Male	5,904 (58.00%)	5,649 (55.50%)	5,106 (50.16%)	
Ethnicity, *n* (%)				<0.001
Caucasian	8,092 (79.50%)	8,304 (81.58%)	8,346 (81.99%)	
African American	1,133 (11.13%)	1,056 (10.37%)	906 (8.90%)	
Hispanic	395 (3.88%)	381 (3.74%)	381 (3.74%)	
Asian	215 (2.11%)	121 (1.19%)	219 (2.15%)	
Native American	99 (0.97%)	80 (0.79%)	77 (0.76%)	
Other/Unknown	245 (2.41%)	237 (2.33%)	250 (2.46%)	
Scr (mg/dL)	1.10 (0.74–1.81)	1.02 (0.72–1.62)	1.15 (0.76–1.90)	<0.001
eGRF	80.00 ± 64.99	85.24 ± 66.28	75.92 ± 55.89	<0.001
BNP (pg/mL)	612.30 (228.00–1662.35)	634.20 (229.00–1684.25)	603.25 (231.25–1672.10)	0.597
COPD, *n* (%)				<0.001
No	9,192 (90.30%)	9,354 (91.90%)	9,358 (91.93%)	
Yes	987 (9.70%)	825 (8.10%)	821 (8.07%)	
HF, *n* (%)				
No	8,438 (82.90%)	8,956 (87.99%)	8,925 (87.68%)	
Yes	1,741 (17.1%)	1,223 (12.01%)	1,254 (12.32%)	
AMI, *n* (%)				<0.001
No	9,597 (94.28%)	9,700 (95.29%)	9,660 (94.90%)	
Yes	582 (5.72%)	479 (4.71%)	519 (5.10%)	
DM, *n* (%)				0.006
No	9,116 (89.56%)	9,236 (90.74%)	9,229 (90.67%)	
Yes	1,063 (10.44%)	943 (9.26%)	950 (9.33%)	
TI, *n* (%)				<0.001
No	7,207 (70.80%)	8,036 (78.95%)	7,230 (71.03%)	
Yes	2,972 (29.20%)	2,143 (21.05%)	2,949 (28.97%)	
Dialysis, *n* (%)				<0.001
No	9,702 (95.31%)	9,882 (97.08%)	9,831 (96.58%)	
Yes	477 (4.69%)	297 (2.92%)	348 (3.42%)	
ICU LOS, d, median (IQR)	4.00 (2.79–6.90)	3.20 (2.53–4.90)	3.71 (2.74–5.86)	<0.001
Hospital LOS, d, median (IQR)	8.09 (5.20–13.55)	6.21 (4.11–9.72)	7.42 (4.93–11.73)	<0.001
APACHE IV	62.78 ± 25.73	59.24 ± 25.28	66.76 ± 27.24	<0.001
ICU mortality, *n* (%)				<0.001
No	9,726 (95.55%)	10,073 (94.57%)	9,864 (90.37%)	
Yes	453 (4.45%)	578 (5.43%)	1,051 (9.63%)	
Hospital mortality, *n* (%)				<0.001
No	9,312 (91.48%)	9,212 (90.50%)	8,794 (86.39%)	
Yes	867 (8.52%)	967 (9.50%)	1,385 (13.61%)	

Continuous variables are summarized as mean (SD) or median (trisection interval); categorical variables are presented as percentages (%).

BMI, body mass index; COPD, chronic obstructive pulmonary disease; HF, heart-failure; AMI, acute myocardial infarction; DM, diabetes mellitus; ICU, intensive care unit; LOS, length of stay; TI, tracheal intubation; Scr, serum creatinine; eGFR, estimated glomerular filtration rate; BNP, brain natriuretic peptide.

### Feature selection and predictor importance

**Figure 2:**
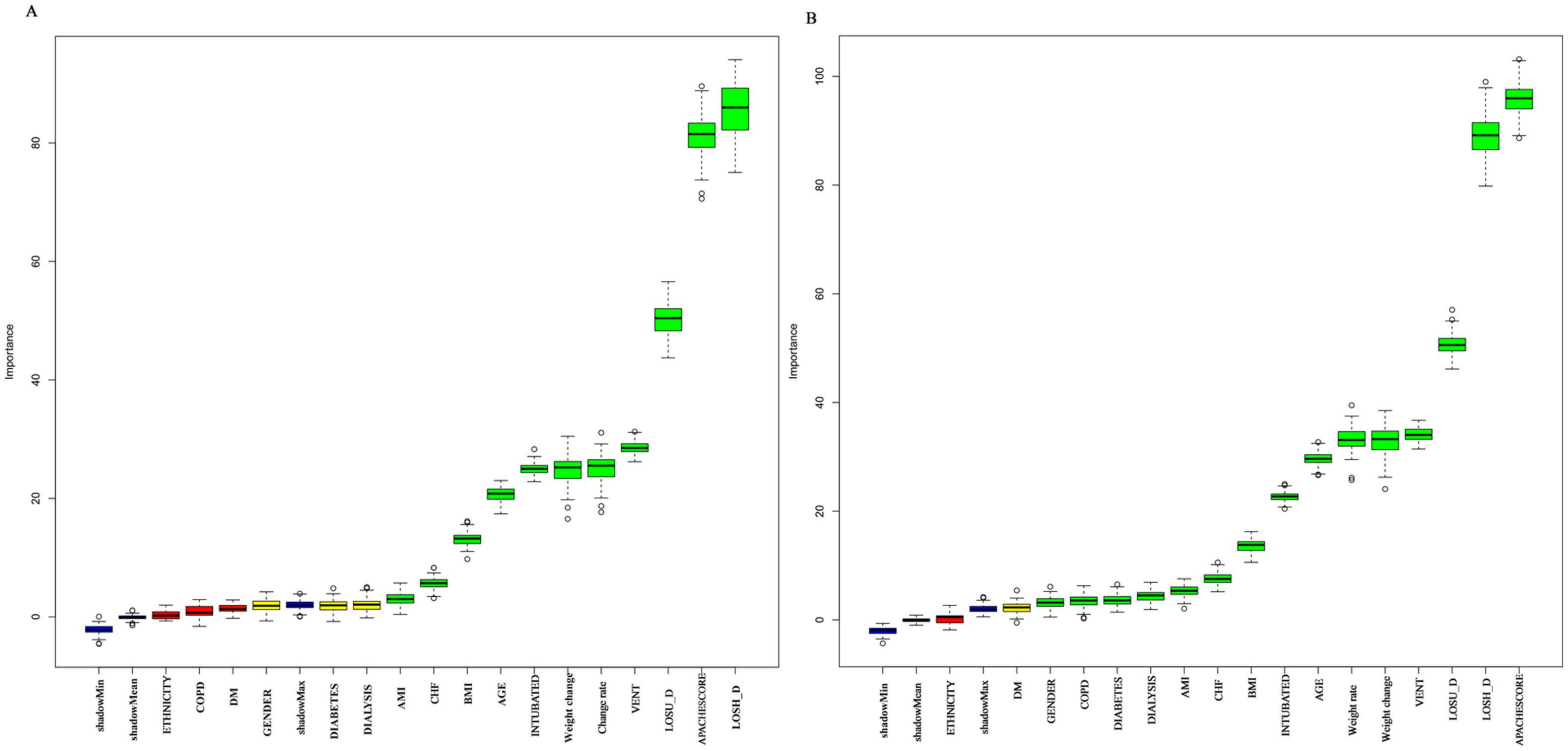
Variable-importance ranking for the mortality-prediction model in critically ill patients (Boruta algorithm). **(A,B)** Show the importance rankings for ICU mortality and hospital mortality, respectively.

We used the Boruta algorithm to identify the variables most strongly associated with mortality (Figure 2). For both ICU and hospital mortality, established severity scores (SOFA, APACHE IV) and interventions such as endotracheal intubation and mechanical ventilation ranked highest. Notably, the “weight-change rate”—the focus of the present study—was ranked 5th for ICU and 6th for hospital death, with importance values well above the shadow-maximum threshold (shadowMax), confirming its independent and robust predictive value.

### Association between body weight change rate and mortality

Univariable analyses (Supplementary Table S1) showed that each additional percentage point increase in weight change rate was significantly associated with higher odds of both ICU mortality (OR 1.05, 95% CI 1.05–1.06) and hospital mortality (OR 1.04, 95% CI 1.04–1.05) (*p* < 0.001). Multivariable logistic regression models were then used to test the independence of these relationships (Table 2). In Model II (adjusted for demographics only), every 1% increase in weight-change rate was associated with 5% higher odds of ICU death (OR 1.05) and 4% higher odds of hospital death (OR 1.04). After full adjustment in Model IV (demographics, APACHE IV, comorbidities, and key interventions), the association remained robust: ICU mortality OR 1.04, 95% CI 1.04–1.05; hospital mortality OR 1.03, 95% CI 1.03–1.04 (all *p* < 0.001).

**Table 2:** Relationship between weight change rate and mortality in critically ill patients in different models.

	Exposure	Model I (OR, 95%CI) *P*	Model II (OR, 95%CI) *P*	Model III (OR, 95%CI) *P*	Model IV (OR, 95%CI) *P*
ICU mortality	Weight change rate (%)	1.05 (1.05, 1.06) < 0.001	1.05 (1.05, 1.06) < 0.001	1.04 (1.03, 1.05) < 0.001	1.04 (1.03, 1.05) < 0.001
	Tertiles				
	T1	Ref	Ref	Ref	Ref
	T2	1.25 (1.11, 1.42) 0.0005	1.26 (1.11, 1.43) 0.0003	1.46 (1.28, 1.67) < 0.0001	1.52 (1.31, 1.76) < 0.0001
	T3	1.93 (1.71, 2.17) < 0.0001	1.95 (1.73, 2.20) < 0.0001	1.74 (1.54, 1.97) < 0.0001	1.77 (1.55, 2.03) < 0.0001
	P for trend	<0.001	<0.001	<0.001	<0.001
Hospital mortality	Weight change rate (%)	1.04 (1.04, 1.05) < 0.001	1.04 (1.04, 1.05) < 0.001	1.03 (1.03, 1.04) < 0.001	1.03 (1.03, 1.04) < 0.001
	Tertiles				
	T1	Ref	Ref	Ref	Ref
	T2	1.13 (1.02, 1.24) 0.014	1.13 (1.02, 1.24) 0.014	1.29 (1.17, 1.43) < 0.001	1.35 (1.20, 1.51) < 0.0001
	T3	1.69 (1.55, 1.85) < 0.001	1.68 (1.54, 1.84) < 0.001	1.53 (1.39, 1.68) < 0.001	1.60 (1.44, 1.78) < 0.0001
	P for trend	<0.001	<0.001	<0.001	<0.001

Model I: we did not adjust other covariates.

Model II: we adjusted gender, age, ethnicity, BMI.

Model III: we adjusted gender, age, ethnicity, BMI, APACHE IV score.

Model IV: we adjusted gender, age, ethnicity, BMI, APACHE IV score, COPD, HF, AMI, DM, intubated and dialysis and eGFR. OR, odds ratio; CI, confidence, Ref, reference.

Analyses across tertiles of weight-change rate showed that, relative to T1, patients in T3 (greatest weight gain) carried the highest mortality risk in Model IV: ICU mortality OR 1.77, 95% CI 1.55–2.03; hospital mortality OR 1.60, 95% CI 1.44–1.78. Test for trend confirmed a progressive increase in risk with greater weight-change rate (P for trend < 0.001). Supplementary Figure S2 illustrates the stepwise rise in both ICU and hospital mortality across increasing tertiles.

### Non-linear relationship and threshold effect

**Figure 3:**
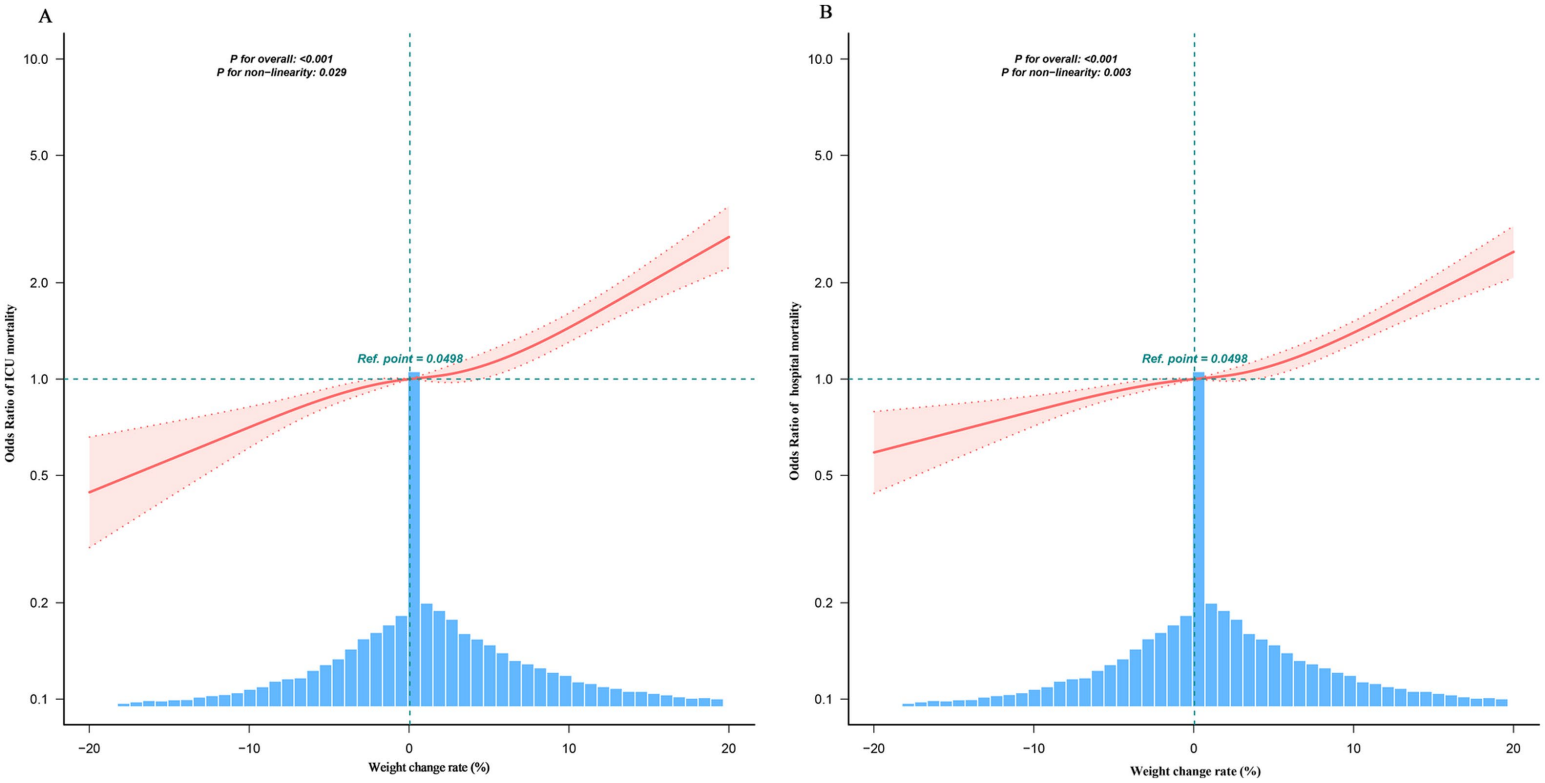
Relationship between weight change rate and ICU or hospital mortality in critically ill patients. Using a generalized additive model adjusted for gender, age, ethnicity, BMI, APACHE IV score, COPD, HF, AMI and DM, IT, eGFR and dialysis, a threshold–non-linear association was identified: both ICU **(A)** and hospital **(B)** mortality remained low until approximately 5% weight loss, then rose sharply with further weight loss (solid red line; light-red shaded area = 95% CI).

Generalized additive models (GAM) revealed a non-linear association between body weight change rate and mortality (Figures 3A,B). The risk of death remained relatively stable at lower magnitudes of weight change, but increased markedly once the change exceeded a critical threshold.

Accordingly, we fitted two-segment linear regression models to quantify this threshold (Table 3). For ICU mortality, the turning point (knot, K) was identified at a weight-change rate of 5.14%. For weight change below 5.14%, each additional 1% increase in the rate was associated with a 3% increase in the odds of ICU mortality (OR 1.03, 95% CI 1.02–1.04, *p* < 0.001). Above this threshold (≥5.14%), each 1% increase conferred 6% higher odds of ICU death (OR 1.06, 95% CI 1.04–1.08, *p* < 0.001). A similar breakpoint was observed for hospital mortality at 4.73%. Likelihood-ratio tests confirmed that the piecewise linear model fitted the data significantly better than the simple linear model (*p* ≤ 0.01), validating the presence of a non-linear threshold effect.

**Table 3:** Threshold effect analysis of the weight change rate and mortality.

Models	ICU mortality		Hospital mortality	
	OR (95% CI)	*P* value	OR (95% CI)	*P* value
Model I				
One line effect	1.04 (1.03, 1.05)	<0.001	1.03 (1.03, 1.04)	<0.001
Model II				
Turning point (K)	5.14		4.73	
Weight change rate <K	1.03 (1.02, 1.04)	<0.001	1.02 (1.01, 1.03)	<0.001
Weight change rate ≥K	1.06 (1.04, 1.08)	<0.001	1.05 (1.04, 1.06)	<0.001
*P* value for LRT test*	0.029		0.010	

Data were presented as OR (95% CI) *P* value; Model I, linear analysis; Model II, non-linear analysis. Adjusted for gender, age, ethnicity, BMI, APACHE IV score, COPD, HF, AMI, and DM, intubation, eGFR and dialysis. CI, confidence interval; OR, odds ratio; LRT, logarithm likelihood ratio test. * *p* < 0.05 indicates that model II is significantly different from Model I.

### Subgroup analyses

We conducted extensive subgroup analyses to examine the robustness of the association between body weight change rate and ICU mortality (Supplementary Table S2) as well as hospital mortality (Supplementary Table S3). The results showed that the positive relationship between increasing weight change rate and higher mortality risk remained consistent across the vast majority of pre-specified subgroups.

### Predictive performance comparison

Finally, we compared the discriminative ability of different weight-related indices for predicting mortality (Supplementary Table S4). Among all metrics, the weight-change rate demonstrated the highest predictive performance, with an AUC of 0.588 (95% CI: 0.573–0.602) for ICU mortality and 0.572 (95% CI: 0.563–0.583) for hospital mortality, which were numerically higher than those of admission weight, discharge weight, or admission BMI alone.

### Correlation analysis of BNP with prognosis in ICU patients

In the subgroup of 2,218 patients (7.26%) with BNP data, BNP levels positively correlated with weight change rate (r = 0.12, *p* < 0.001) (Supplementary Figure S3), suggesting utility in distinguishing fluid retention from nutritional changes. Stratified at 600 pg./mL, the high-BNP group (*n* = 1,125) exhibited significantly higher ICU mortality than the low-BNP group (*n* = 1,093) (7.29% vs. 4.03%) (Supplementary Figure S4), and median BNP was significantly higher in non-survivors (858.5 vs. 598.1 pg./mL, *p* < 0.001) (Supplementary Table S5).

## Discussion

Our retrospective analysis demonstrates that in-ICU body weight change rate exhibits a positive, non-linear association with mortality and establishes its independent prognostic value. The key findings are as follows: (1) Each 1% increase in the weight-change rate was independently associated with higher mortality—after full adjustment, the OR for ICU death was 1.04 (95% CI 1.03–1.05) and for hospital death was 1.03 (95% CI 1.03–1.04); (2) A threshold effect was detected at approximately 5% (inflection point: 5.14% for ICU mortality and 4.73% for hospital mortality), beyond which the risk increased more steeply; and (3) The weight-change rate showed the highest predictive performance, with an AUC of 0.588 for ICU mortality and 0.572 for hospital mortality—values that were numerically higher than those of static indices such as admission weight, discharge weight, or admission BMI.

### Independence and novelty of weight change rate as a prognostic indicator

Using Boruta feature selection and multivariable modeling, we demonstrated that the weight change rate provides prognostic information that complements traditional severity scores (APACHE IV, SOFA). This contrasts with earlier work focused on static anthropometry. For instance, Pieracci et al. found no association between admission BMI and mortality of surgical critical illness (16), whereas Zusman et al. highlighted the poor stability of single-point nutritional markers (8). Although the clinical context in adult ICUs is far more complex, this physiological rationale indirectly supports the concept that short-term weight variation may be strongly influenced by fluid shifts. Previous studies in adults have reported associations between weight or BMI change and clinical outcomes (9, 10), but did not examine potential non-linearity or employ machine-learning-based feature selection. Ayalon et al. focused on static weight in pediatric critical illness (11). Our study extends this evidence by confirming the independent prognostic value of the weight-change rate in a large adult cohort and, to our knowledge, is the first to characterize its non-linear relationship with mortality.

### Clinical implications of the non-linear relationship and threshold

The generalized additive model and two-segment regression revealed a non-linear association: mortality risk remained relatively stable at lower magnitudes of weight change but increased markedly once the change exceeded a critical rate of approximately 5% (ICU: 5.14%; hospital: 4.73%). This pattern is consistent with earlier fluid-balance research. For example, Payen et al. reported that a positive cumulative fluid balance was an important factor associated with increased 60-day mortality, supporting the link between fluid overload and adverse outcomes (17). The inflection point around +5% weight change may represent a transition from a compensated to a decompensated physiological state, providing a clinically actionable warning threshold.

### Discriminative power and bedside utility

ROC analysis demonstrated that the weight-change rate achieved AUCs of 0.588 for ICU mortality and 0.572 for hospital mortality. These values were numerically higher than those of static indices such as admission weight, discharge weight, or admission BMI. A systematic review by Aminiahidash et al. reported that existing ICU nutrition assessment tools commonly yield AUCs in the range of 0.55–0.65 (18). Our metric reaches the upper end of this performance spectrum without requiring specialized tests, aligning well with established criteria for a useful bedside tool: it is practical, objective, and sensitive. Looking ahead, integrating serial weight-change trends with dynamic severity scores (e.g., SOFA) or inflammatory biomarkers could further enhance the accuracy of multivariable prediction models in critical care.

### The association is plausibly driven by several intertwined mechanisms

First, pronounced weight gain largely reflects positive fluid balance and overload, which impair tissue oxygenation, increase diffusion distances, and compromise—especially cardiac and pulmonary—organ function, ultimately raising mortality (5). Second, rapid weight loss may indicate severe catabolism and muscle wasting that underlie ICU-acquired weakness, prolonged weaning, extended LOS, and long-term death (2, 6). Although we could not partition fluid from lean-mass changes, Sundström et al. showed that dynamic weight variation itself integrates metabolic stress and nutritional state (14).

### Natriuretic peptides as adjuncts for interpreting weight changes

Critically, B-type Natriuretic Peptide (BNP) and NT-proBNP may help distinguish fluid-related weight changes from nutritional alterations. In our cohort, BNP correlated with weight change rate (r = 0.12, *p* < 0.001), and patients with BNP > 600 pg./mL showed higher ICU mortality (7.29% vs. 4.03%). This threshold aligns with Di Marca et al. (19), where BNP > 600 pg./mL combined with malnutrition and CKD predicted 30-day mortality (OR = 2.70) and 3-month re-hospitalization (OR = 12.28). Similarly, Granata et al. (20) demonstrated BNP identifies asymptomatic heart failure and explains weight fluctuations. In dialysis patients, natriuretic peptides predict survival (21), ventricular remodeling (22), and remain prognostic in volume-expanded states (23). Thus, integrating BNP monitoring with weight measurements could enhance risk stratification around the 5% threshold, distinguishing fluid overload (high BNP) from catabolism (low BNP) to guide targeted therapy.

### Strengths and limitations

This study has several notable strengths. First, by utilizing the eICU Collaborative Research Database, which encompasses >200 ICUs and >200,000 admissions across the United States, we assembled a racially, age-wise, and clinically diverse analytic cohort of 30,537 critically ill patients. This large, multicenter sample enhances the external validity and statistical power of our findings. Second, we employed the Boruta machine-learning algorithm for robust feature selection. By comparing the importance of real predictors against permuted “shadow” variables, this method objectively identified body weight change rate as an informative prognostic factor independent of traditional severity scores (e.g., APACHE IV), thereby mitigating the subjectivity inherent in conventional stepwise regression approaches.

Several limitations should also be acknowledged. First, the retrospective observational design precludes causal inference. Although we adhered to STROBE guidelines and extensively adjusted for known confounders, residual bias from unmeasured factors (e.g., detailed diuretic use, daily fluid balance nuances) may persist. Second, the eICU-CRD only provides admission and discharge weights, lacking daily weight trajectories. This, combined with the absence of gold-standard body composition measures (e.g., bioimpedance, CT-derived muscle area), means we could not differentiate the specific contributions of fluid overload versus lean-tissue loss to mortality. Furthermore, we could not account for extreme clinical conditions (e.g., anasarca, amputation, or pregnancy) that might distort weight readings. Third, the application of stringent quality filters excluded a substantial fraction of records—chiefly short-stay admissions and those with missing key variables—potentially limiting generalizability to early-transfer or data-sparse populations. Fourth, detailed data on nutritional support (e.g., caloric/protein delivery, route of administration) are unavailable, limiting exploration of the causal pathway linking weight change to nutritional interventions and outcomes. Fifth, as noted, the lack of body composition data precludes determining whether fat-free mass depletion or volume expansion is the dominant driver of the observed risk. Future prospective studies incorporating serial body composition measurements (e.g., via CT, bioimpedance, or DXA) and detailed nutritional data are warranted to clarify the underlying mechanisms and clinical utility of monitoring weight change in critically ill patients. Finally, BNP data were available in only a minority of patients (*n* = 2,218), limiting the statistical power to definitively establish BNP-guided risk stratification for weight change management. Future prospective studies with protocolized BNP/NT-proBNP monitoring are warranted to validate these preliminary associations.

## Conclusion

In summary, dynamic monitoring of body weight change rate during ICU stay serves as an independent, non-linear predictor of in-hospital mortality in critically ill patients, demonstrating superior discriminative ability compared to static indices such as admission weight or BMI. Although mortality risk increases across the spectrum of weight change, the slope of this association steepens markedly beyond a threshold of approximately 5%, providing a clinically actionable inflection point for early risk stratification. These findings support the integration of weight-change rate into routine clinical surveillance to identify high-risk patients and guide timely, personalized interventions. Future prospective studies are warranted to validate these observations and to investigate whether therapeutic strategies aimed at modulating weight-change trajectories can improve patient-centered outcomes.

## Data Availability

Publicly available datasets were analyzed in this study. This data can be found via the eICU Collaborative Research Database at https://eicu-crd.mit.edu/.
