# Supplementary material for: Weight changes and all-cause mortality in critically ill patients: a multi-center retrospective cohort study
Source: Front Nutr. 2026 May 11;13:1722116. doi: 10.3389/fnut.2026.1722116 (PMC13198983; doi:10.3389/fnut.2026.1722116)
Supplement: Supplementary file 1 [file Table_1.docx]

# Weight changes and all-cause mortality in critically ill patients: a multi-center retrospective cohort study

Cheng Luo^1^, Tinglong Zhang^2^, Zhenhua Huang^1^, Xiaoyong Xiao^1,3 *^ and Zhe Deng ^1*^

1 Department of Emergency Medicine, the First Affiliated Hospital of Shenzhen University, Shenzhen Second People’s Hospital, Shenzhen, 518035, China.

2 Department of Emergency Medicine, South China Hospital Affiliated to Shenzhen University, Shenzhen, 518111, China.

3. Liuzhou Key Laboratory of Emergency and Critical Care Medical Research, Liuzhou 545006, China

Cheng Luo, Zhenhua Huang and Tinglong Zhang contributed equally to this work.

*Corresponding authors:

Xiaoyong Xiao, Department of Emergency Medicine, the First Affiliated Hospital of Shenzhen University, Shenzhen Second People’s Hospital, Shenzhen, 518035, China & The First Affiliated Hospital of Jinan University, Guangzhou, China

email: as575947548@126.com.

Zhe Deng, Department of Emergency Medicine, the First Affiliated Hospital of Shenzhen University, Shenzhen Second People’s Hospital, Shenzhen, 518035, China & The First Affiliated Hospital of Jinan University, Guangzhou, China

email: [dengz163@163.com](mailto:dengz163@163.com).

TABLE S1 Factors influencing risk of ICU and hospital mortality analyzed by univariate analysis

| Exposure | Statistics | ICU mortality  OR (95% CI) P-value | Hospital mortality  OR (95% CI) P-value |
| --- | --- | --- | --- |
| Age (year) | 64.10 ± 15.99 | 1.01 (1.01, 1.01) <0.001 | 1.02 (1.02, 1.02) <0.001 |
| BMI (kg/m2) | 29.77 ± 8.66 | 1.00 (1.00, 1.01) 0.629 | 1.00 (0.99, 1.00) 0.046 |
| Gender |  |  |  |
| Female, n (%) | 13,878 (45.45%) | 1.0 | 1.0 |
| Male, n (%) | 16,659 (54.55%) | 1.15 (1.04, 1.26) 0.005 | 1.10 (1.02, 1.18) 0.012 |
| **Ethnicity** |  |  |  |
| Caucasian | 24,742 (81.02%) | 1.0 | 1.0 |
| African American | 3,095 (10.14%) | 0.83 (0.70, 0.98) 0.026 | 0.93 (0.82, 1.05) 0.229 |
| Hispanic | 1,157 (3.79%) | 1.05 (0.83, 1.34) 0.676 | 0.89 (0.73, 1.08) 0.237 |
| Asian | 555 (1.82%) | 0.81 (0.55, 1.18) 0.272 | 0.97 (0.74, 1.28) 0.856 |
| Native American | 256 (0.84%) | 1.29 (0.81, 2.04) 0.282 | 1.11 (0.76, 1.63) 0.596 |
| Other/Unknown | 732 (2.40%) | 0.90 (0.65, 1.24) 0.521 | 0.83 (0.64, 1.07) 0.148 |
| Scr (mg/dL) | 1.09 (0.74-1.79) | 1.12 (1.10, 1.14) <0.001 | 1.11 (1.09, 1.13) <0.001 |
| eGRF | 68.51 (38.16-108.94) | 0.99 (0.99, 0.99) <0.001 | 0.99 (0.99, 0.99) <0.001 |
| No, n (%) | 27,904 (91.38%) | 1.0 | 1.0 |
| Yes, n (%) | 2,633 (8.62%) | 0.91 (0.76, 1.08) 0.275 | 1.08 (0.95, 1.22) 0.246 |
| **HF** |  |  |  |
| No, n (%) | 26319 (86.19%) | 1.0 | 1.0 |
| Yes, n (%) | 4218 (13.81%) | 1.22 (1.07, 1.38) <0.001 | 1.25 (1.13, 1.38) <0.001 |
| **AMI** |  |  |  |
| No, n (%) | 28,957 (94.83%) | 1.0 | 1.0 |
| Yes, n (%) | 1,580 (5.17%) | 1.51 (1.26, 1.81) <0.001 | 1.35 (1.16, 1.56) <0.001 |
| **DM** |  |  |  |
| No, n (%) | 27,581 (90.32%) | 1.0 | 1.0 |
| Yes, n (%) | 2,956 (9.68%) | 0.89 (0.76, 1.05) 0.184 | 1.04 (0.92, 1.18) 0.512 |
| **TI** |  |  |  |
| No, n (%) | 22,473 (73.59%) | 1.0 | 1.0 |
| Yes, n (%) | 8,064 (26.41%) | 2.93 (2.67, 3.22) <0.001 | 2.28 (2.11, 2.45) <0.001 |
| **Dialysis** |  |  |  |
| No, n (%) | 29,415 (96.33%) | 1.0 | 1.0 |
| Yes, n (%) | 1,122 (3.67%) | 1.54 (1.25, 1.91) <0.001 | 1.47 (1.24, 1.74) <0.001 |
| Hospital LOS, d, median (IQR) | 7.17 (4.77-11.64) | 0.93 (0.92, 0.94) <0.001 | 0.99 (0.99, 1.00) 0.001 |
| ICU LOS, d, median (IQR) | 3.65 (2.68-5.85) | 1.05 (1.04, 1.05) <0.001 | 1.05 (1.04, 1.05) <0.001 |
| Weight change rate (%) | 0.92 ± 6.73 | 1.05 (1.05, 1.06) <0.001 | 1.04 (1.04, 1.05) <0.001 |
| APACHE IV | 62.93 ± 26.28 | 1.04 (1.03, 1.04) <0.001 | 1.03 (1.03, 1.03) <0.001 |

BMI, body mass index; COPD, chronic obstructive pulmonary disease; CHF, congestive heart-failure; AMI, acute myocardial infarction; DM, diabetes mellitus; ICU, intensive care unit; LOS, length of stay; Scr, serum creatinine; eGFR, estimated glomerular filtration rate.

**Table S2** Stratified associations between weight change and ICU mortality by age, gender, BMI, ICU type, ethnicity, hospital discharge year and APACHE IV.

| **Variable** | **Number** | **OR (95% CI)** | **P** |
| --- | --- | --- | --- |
| **Age, yeas, Tertile** |  |  |  |
| T1 | 10,107 | 1.06 (1.05, 1.08) | <0.001 |
| T2 | 10,172 | 1.04 (1.03, 1.05) | <0.001 |
| T3 | 10,258 | 1.03 (1.02, 1.04) | <0.001 |
| **BMI (kg/m 2), Tertile** |  |  |  |
| T1 | 10,178 | 1.03 (1.02, 1.04) | <0.001 |
| T2 | 10,173 | 1.05 (1.03, 1.06) | <0.001 |
| T3 | 10,186 | 1.04 (1.03, 1.06) | <0.001 |
| **Gender** |  |  |  |
| Female | 13,878 | 1.04 (1.03, 1.05) | <0.001 |
| Male | 16,659 | 1.04 (1.03, 1.05) | <0.001 |
| **Ethnicity** |  |  |  |
| Caucasian | 24,742 | 1.04 (1.03, 1.05) | <0.001 |
| African American | 3,095 | 1.05 (1.02, 1.07) | <0.001 |
| Hispanic | 1,157 | 1.05 (1.01, 1.08) | 0.008 |
| Asian | 555 | 1.05 (1.00, 1.11) | 0.075 |
| Native American | 256 | 1.02 (0.95, 1.09) | 0.618 |
| Other/Unknown | 732 | 1.10 (1.04, 1.16) | <0.001 |
| **Hospital discharge year** |  |  |  |
| 2014 | 14,378 | 1.04 (1.03, 1.05) | <0.001 |
| 2015 | 16,159 | 1.04 (1.03, 1.05) | <0.001 |
| **APACHE IV, Tertile** |  |  |  |
| T1 | 9,942 | 1.05 (1.02, 1.08) | <0.001 |
| T2 | 10,041 | 1.05 (1.03, 1.06) | <0.001 |
| T3 | 10,554 | 1.05 (1.04, 1.05) | <0.001 |

Above model adjusted for gender, age, ethnicity, BMI, APACHE IV score, medical history (COPD, CHF, AMI and DM), intubated and dialysis. In each case, the model is not adjusted for the stratification variable when the stratification variable was a categorical variable.

**Table S3** Stratified associations between weight change and hospital mortality by age, gender, BMI, ICU type, ethnicity, hospital discharge year and APACHE IV.

| **Variable** | **Number** | **OR (95% CI)** | **P** |
| --- | --- | --- | --- |
| **Age, yeas, Tertile** |  |  |  |
| T1 | 10,107 | 1.05 (1.04, 1.06) | <0.001 |
| T2 | 10,172 | 1.03 (1.02, 1.04) | <0.001 |
| T3 | 10,258 | 1.02 (1.01, 1.03) | <0.001 |
| **BMI (kg/m 2), Tertile** |  |  |  |
| T1 | 10,178 | 1.03 (1.02, 1.04) | <0.001 |
| T2 | 10,173 | 1.04 (1.03, 1.05) | <0.001 |
| T3 | 10,186 | 1.03 (1.02, 1.04) | <0.001 |
| **Gender** |  |  |  |
| Female | 13,878 | 1.03 (1.02, 1.04) | <0.001 |
| Male | 16,659 | 1.03 (1.03, 1.04) | <0.001 |
| **Ethnicity** |  |  |  |
| Caucasian | 24,742 | 1.03 (1.03, 1.04) | <0.001 |
| African American | 3,095 | 1.04 (1.02, 1.06) | <0.001 |
| Hispanic | 1,157 | 1.04 (1.01, 1.07) | 0.0120 |
| Asian | 555 | 1.06 (1.01, 1.10) | 0.007 |
| Native American | 256 | 1.02 (0.97, 1.08) | 0.411 |
| Other/Unknown | 732 | 1.03 (0.99, 1.07) | 0.172 |
| **Hospital discharge year** |  |  |  |
| 2014 | 14,378 | 1.04 (1.03, 1.05) | <0.001 |
| 2015 | 16,159 | 1.03 (1.02, 1.04) | <0.001 |
| **APACHE IV, Tertile** |  |  |  |
| T1 | 9,942 | 1.04 (1.02, 1.06) | <0.001 |
| T2 | 10,041 | 1.03 (1.02, 1.04) | <0.001 |
| T3 | 10,554 | 1.04 (1.03, 1.05) | <0.001 |

Above model adjusted for gender, age, ethnicity, BMI, APACHE IV score, medical history (COPD, CHF, AMI and DM), intubated and dialysis. In each case, the model is not adjusted for the stratification variable when the stratification variable was a categorical variable.

**Table S4** **Discriminative Ability of weight indices for Mortality for critical ill patients**

| Variable | ICU mortality | | Hospital mortality | |
| --- | --- | --- | --- | --- |
|  | AUC | (95%CI) | AUC | (95%CI) |
| Admission weight (kg) | 0.500 | 0.486-0.514 | 0.517 | 0.506-0.527 |
| Discharge weight (kg) | 0.523 | 0.509-0.537 | 0.502 | 0.491-0.513 |
| Admission BMI (kg/m2) | 0.505 | 0.491-0.518 | 0.518 | 0.507-0.529 |
| Weight change rate (%) | 0.588 | 0.573-0.602 | 0.572 | 0.563-0.583 |

**Table S5 Comparison of BNP levels across ICU discharge status groups.**

| ICU discharge status | Alive | Death | P value |
| --- | --- | --- | --- |
| N | 2092 | 126 |  |
| BNP | 598.05 (236.00-1494.25) | 858.50 (435.50-2435.43) | <0.001 |


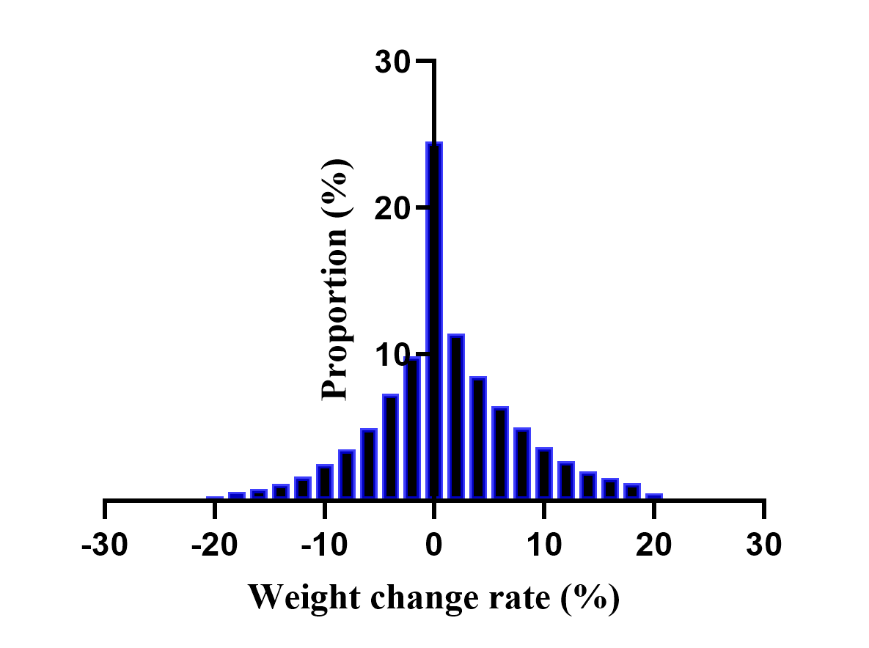


Figure S1. Distribution of weight change rate (%). It presented a normal distribution, ranging from -19.98% to 20.00%, with a mean of 0.92.


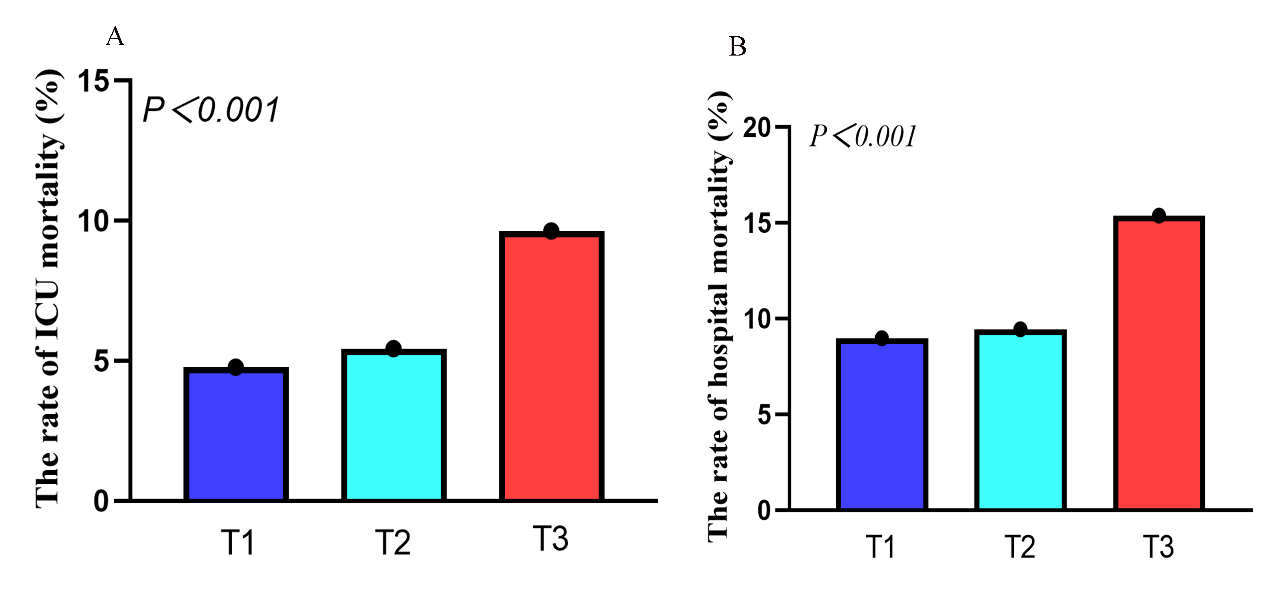


Figure S2. the Relationship Between weight change rate tertiles and the rate of ICU or hospital mortality.


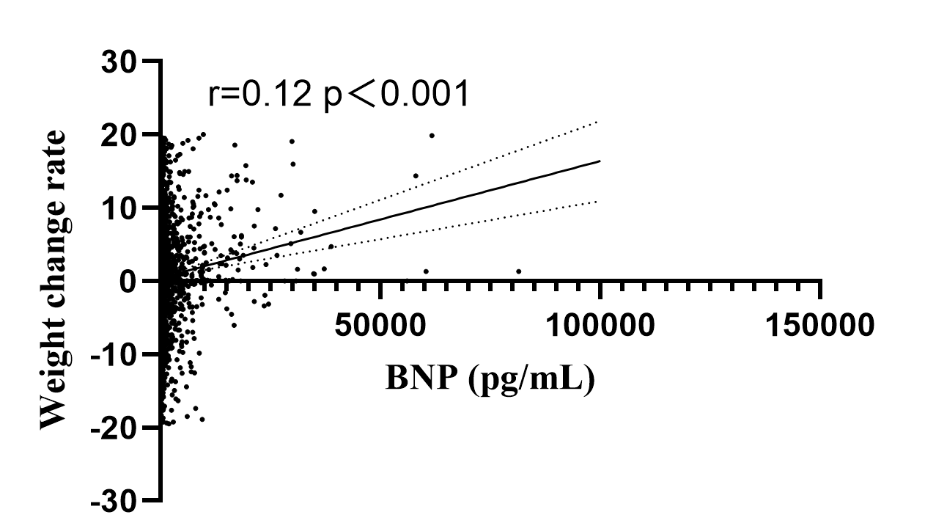


Figure S3. Correlation between weight change rate and BNP.


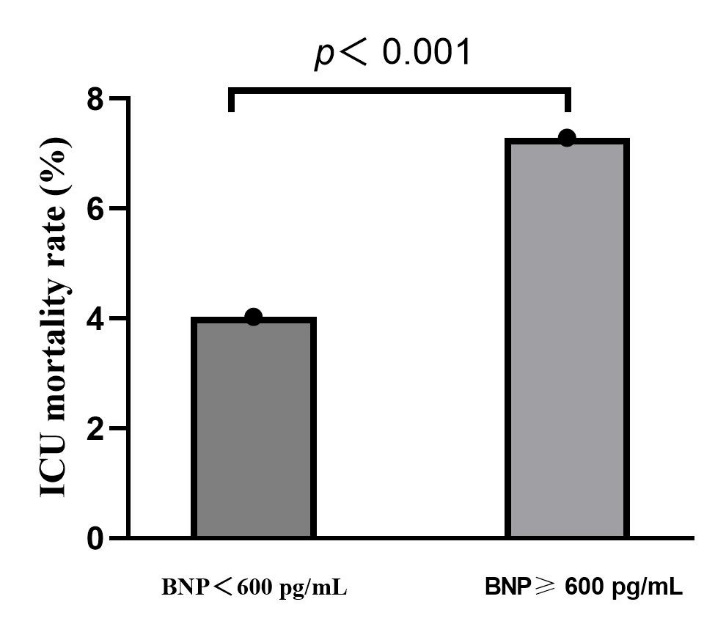


Figure S4. ICU mortality stratified by BNP levels (≥600 vs. <600 pg/mL).
